# Deep Brain Stimulation in Parkinson's Disease: New and Emerging Targets for Refractory Motor and Nonmotor Symptoms

**DOI:** 10.1155/2017/5124328

**Published:** 2017-07-06

**Authors:** Dustin Anderson, Grayson Beecher, Fang Ba

**Affiliations:** Department of Medicine, University of Alberta, Edmonton, AB, Canada T6G 2G3

## Abstract

Parkinson's disease (PD) is a progressive neurodegenerative condition characterized by bradykinesia, tremor, rigidity, and postural instability (PI), in addition to numerous nonmotor manifestations. Many pharmacological therapies now exist to successfully treat PD motor symptoms; however, as the disease progresses, it often becomes challenging to treat with medications alone. Deep brain stimulation (DBS) has become a crucial player in PD treatment, particularly for patients who have disabling motor complications from medical treatment. Well-established DBS targets include the subthalamic nucleus (STN), the globus pallidus pars interna (GPi), and to a lesser degree the ventral intermediate nucleus (VIM) of the thalamus. Studies of alternative DBS targets for PD are ongoing, the majority of which have shown some clinical benefit; however, more carefully designed and controlled studies are needed. In the present review, we discuss the role of these new and emerging DBS targets in treating refractory axial motor symptoms and other motor and nonmotor symptoms (NMS).

## 1. Introduction

Parkinson's disease (PD) is a common neurodegenerative condition. Many successful pharmacological therapies and strategies have been developed to treat both the motor and nonmotor manifestations of PD; however, as PD progresses it often becomes intractably difficult to treat, typically as a result of motor complications related to treatment. Since the seminal study by Benabid et al. targeting the ventral intermediate nucleus (VIM) of the thalamus [1], deep brain stimulation (DBS) has emerged as a key player in the treatment of PD. Multiple randomized controlled studies have demonstrated subthalamic nucleus- (STN-) and globus pallidus interna- (GPi-) DBS to be superior to medical treatment alone in treating a number of the cardinal symptoms and motor complications from therapy [1–3]. The benefit of DBS on axial symptoms is less clear. Several reports have indicated improvement of posture, gait, and balance control after STN- or GPi-DBS, when these symptoms were responsive to levodopa treatment before DBS surgery [4–9]; however, the benefit on postural instability (PI) and gait is not sustained [4]. Moreover, it has been noted that a significant number of patients report postoperative worsening of gait, despite concurrent improvement in motor scores and global outcomes after bilateral STN-DBS. Further, fall risk has been demonstrated to increase and levodopa-resistant freezing of gait (FoG) persists or worsens [10–16]. The axial domains of speech [17–19] and swallowing [20, 21] have also shown to be impacted by DBS. To complicate matters further, stimulation parameters (i.e., high frequency stimulation) can also lead to adverse axial effects in patients. These disparities in outcome have fueled the exploration for novel DBS targets that may prove beneficial at treating the axial motor symptoms of PD. In addition to refractory axial motor symptoms, it is clear that nonmotor symptoms (NMS) can also become particularly troublesome [22], as PD progresses and increases in severity. NMS have a significant impact on prognosis and quality of life [23], again highlighting the need for alternative DBS targets that will have therapeutic benefit not only for refractory motor symptoms, but for NMS in PD as well.

**Figure 1:**
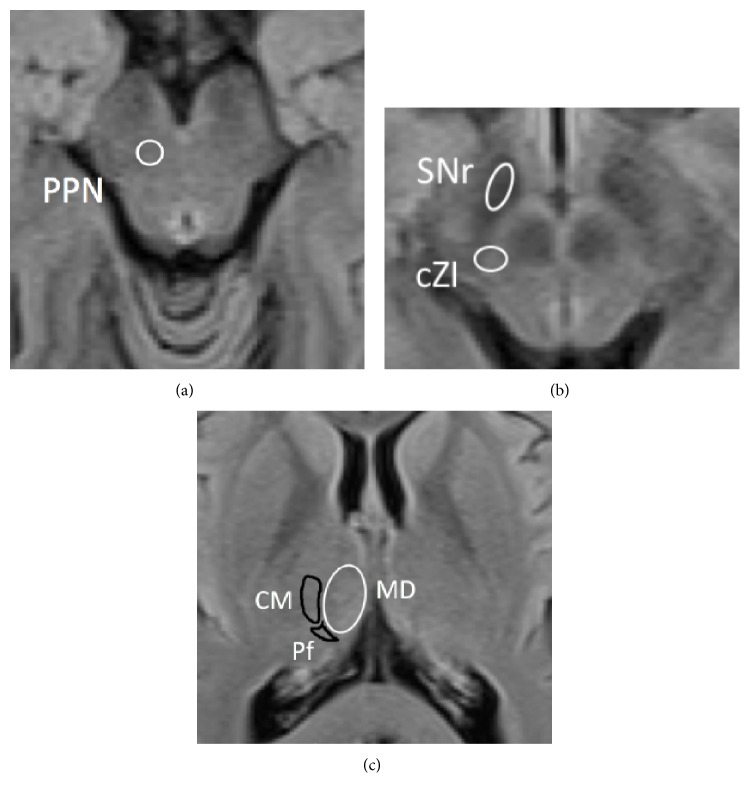
Axial MRI imaging at the level of the midbrain and thalamus, demonstrating the anatomical locations of DBS targets described in the review. CMPf, centromedian-parafascicular nuclear complex; cZI, caudal zona incerta; PPN, pedunculopontine nucleus; SNr, substantia nigra pars reticulata.

In the present review, we discuss new and emerging DBS targets currently being investigated for the treatment of refractory motor symptoms and NMS in PD. These targets include the pedunculopontine nucleus (PPN), the caudal zona incerta (ZI), the substantia nigra (SN) pars reticulate (SNr) (Figure 1), the motor cortex, and other less explored targets.

## 2. New and Emerging DBS Targets for Refractory Motor Symptoms

### 2.1. Refractory Tremor

For tremor-dominant PD, where severe and disabling tremor is refractory to treatment, VIM-DBS has been shown to suppress tremor effectively. In addition, STN- and GPi-DBS both provide sustained benefit for PD resting tremor. For severe tremor and coexisting essential tremor, DBS leads implanted in the posterior aspect of the GPi or STN (i.e., ZI region bordering the STN) appear to be of benefit [23].

#### 2.1.1. Caudal Zona Incerta

The ZI is a small heterogeneous cellular nucleus that lies within the anatomical location termed the posterior subthalamic area (PSA) [24, 25]. The borders of the PSA include the posterior border of the STN anteriorly, the dorsal SN inferiorly, the ventral thalamic nuclei superiorly, the anterolateral red nucleus posteromedially, the medial lemniscus posteriorly, and the internal capsule laterally [24, 25]. The rostral ZI lies along the dorsal and medial STN, while the caudal ZI (cZI) is located posteromedially to the STN [26] (Figure 1(b)). Various functions of the ZI have been postulated throughout the literature; however, it is commonly held that the ZI plays a role in visceral function, arousal, attention, and posture and locomotion, with the cZI mediating the latter [26]. The cZI has widespread afferent and efferent projections amongst the cerebral cortex, diencephalon, brainstem, cerebellum, and spinal cord, the majority of which are GABAergic [26]. While its circuitry remains complex and poorly understood, it is postulated that the cZI may act as an integrator within and between the basal ganglia-thalamocortical loop and the cerebellothalamocortical loop, assisting in the synchronization of oscillatory neuronal firing in both of these pathways [27]. Abnormalities in oscillatory neuronal synchronization that are generated along either of these loops or at the level of the cZI are thought to play a major role in the generation of tremor [24, 25, 27].

The benefit of cZI-DBS for tremor control has been well established in studies investigating its role in essential tremor [28]. In PD, the majority of information that has been gleaned regarding the cZI has come from lesional studies. It has previously been shown that subthalamotomy including the region of the ZI can lead to clinical improvement in PD [29]. Subsequent work focusing on the ZI and the cZI has led to significant discoveries regarding the promise of this structure as a DBS target in PD [24, 25]. The relevance of the cZI as a DBS target in PD was brought to the forefront by Plaha et al., in their study comparing motor outcomes amongst three DBS targets: the cZI, the posterodorsal STN, and dorsomedial/medial STN [30]. When compared to STN stimulation, unilateral cZI stimulation with mean frequency of 150 Hz led to greater improvement in tremor control and overall Unified Parkinson's Disease Rating Scale (UPDRS) motor scores.

A subsequent longitudinal, observational study by Plaha et al. again demonstrated the utility of cZI-DBS (bilateral, 145 Hz) in reducing parkinsonian tremor, as well as a variety of other tremor types, including cerebellar outflow, essential, and dystonic tremor at 12 months of follow-up [27]. Recent work by Blomstedt et al., in an open labeled study with 18 months of follow-up [23–25], echoed the results of Plaha et al. [27], demonstrating the benefit of unilateral cZI-DBS with mean frequency of 160 Hz in the treatment of contralateral, severe parkinsonian tremor. The benefit on rigidity and bradykinesia was not as profound as in STN-DBS; however, a number of studies have suggested that cZI-DBS has a lower incidence of speech deterioration and is associated with better neuropsychological outcomes [27, 31]. That being said, cZI-DBS is not as well established as STN- or GPi-DBS in PD. Further larger scale studies are required to guide future target selection.

#### 2.1.2. Centromedian and Parafascicular Nuclei

The centromedian and parafascicular nuclei (CMPf) (Figure 1(c)) are the two main constituents of the intralaminar nucleus of the thalamus and have several connections within the basal ganglia, with projections to the STN, substantia nigra (SN), and GPi [32]. It has been postulated that CMPf-DBS affects other thalamic components [ventralis oralis anterior (VOA) and VIM] whose role in tremor control has been well established [33, 34].

Interest in the CMPf as a DBS target resurfaced following the observation by Krauss et al. that stimulation of CMPf appeared to abolish resting tremor in 1 patient and involuntary choreoathetotic and dyskinetic movements in 2 others [35]. In subsequent reports, it was observed that CMPf stimulation, independent of STN stimulation, led to reduction of tremor-related muscle activity in 2 patients with PD [36, 37]. Additionally, they demonstrated better tremor control compared with STN-DBS alone. Mazzone et al. [38] demonstrated that combination of CMPf- and GPi-DBS reduced UPDRS III scores by 49.9%, a value significantly different when compared to CMPf or GPi stimulation alone. Unfortunately, tremor control was not specified within the study. Further studies should help clarify whether CMPf stimulation is superior to VIM-DBS for tremor control in PD.

### 2.2. Refractory Axial Motor Symptoms-Gait and Balance

FoG, in addition to other gait disturbances such as decreased stride length and gait variability, is associated with increased fall risk in patients with PD [50]. These symptoms are typically refractory to therapy, including STN- and GPi-DBS [51, 52], and are thus a significant source of morbidity in PD [53]. The pathophysiology and neuropathological substrates underlying FoG remain largely unknown. FoG may be due to a failure to adequately scale amplitudes for the intended movement [54] and/or defective motor programming by the supplementary motor area (SMA) and its maintenance by the basal ganglia, leading to a mismatch between intention and automation [54].

#### 2.2.1. Pedunculopontine Nucleus

The mesencephalic locomotor region (MLR) appears critical for normal gait function [61]. The PPN is a key component of the MLR [62] (Figure 1(a)). Widespread projections involving the PPN include direct glutamatergic inputs from the motor cortex and GABAergic inputs from SNr, GPi, STN, and deep nuclei of the cerebellum. Ascending efferent projections target GPi, SN pars compacta [63], and thalamus. Descending efferent projections target pontine and medullary reticular formations, as well as spinal cord structures vital to the control of muscle tone and locomotion. The PPN appears to play a key role in the initiation, acceleration, deceleration, and termination of locomotion through connections to the basal ganglia and higher cortical regions [61]. PPN neuronal loss is evident in PD [64]. Ways to modulate PPN connectivity and activity have proven elusive. Acetylcholinesterase inhibitors may affect the PPN but effects are likely modest.

Jenkinson et al. were the first group to demonstrate the efficacy of PPN-DBS, in a 1-methyl-4-phenyl-1,2,3,6-tetrahydropyridine- (MPTP-) exposed macaque [65]. Following MPTP exposure, unilateral PPN stimulation was equivalent to levodopa in improving motor activity scores [65]. In 2005, 2 case studies were the first to establish the safety and efficacy of PPN-DBS in humans [39, 40] (Table 1), demonstrating improvements in UPDRS motor scores. Subsequently, a study by Plaha and Gill was the first to show the role of PPN-DBS in improving gait dysfunction and PI in PD [40]. Multiple open labeled PPN-DBS studies have demonstrated clinical improvement in patients with PD, although results have been variable [41, 42, 45, 47] (Table 1). Additional open labeled studies from Thevathasan et al. [45, 46, 66, 67] demonstrated that PPN stimulation (20–35 Hz) improved frequency of falls in PD patients with severe FoG and PI during the “on” state [45]. One study showed improvement in gait and falls questionnaire score but not UPDRS III score in 5 patients with PD implanted with bilateral PPN electrodes [46]. The first double-blinded assessment of PPN-DBS was performed by Ferraye and colleagues [43], demonstrating improvement in FoG but not PI or overall UPDRS scores. The lack of improvement in global motor function and axial symptoms, other than FoG, was in opposition to previous studies (Table 1) [39–41, 46, 48]. Moro et al. were the first to investigate the role of unilateral PPN-DBS in a double-blinded study of 6 patients with PD [44]. At study end period (1 year), UPDRS item 13 (falling) showed 75% improvement, with no statistically significant changes in other motor domains. Furthermore, bilateral stimulation proved more effective than unilateral stimulation [48].

The largest study with the longest follow-up of PPN-DBS in PD was reported by Mazzone et al. [49, 68, 69]. A total of 24 patients with PD and 4 with progressive supranuclear palsy (PSP) [49] were followed for a mean follow-up of 3.8 years. At study end period, they demonstrated an improvement in UPDRS III scores and in axial symptoms (UPDRS items 27–30) (off levodopa therapy); however, no difference was detected between the “on” medication and “off” stimulation state and the “off” medication and “on” stimulation state.

Connectivity to and from the MLR/PPN appears critical for normal gait function and is likely a factor in FoG as well. Structural deficits in connectivity are evident between the basal ganglia and PPN, in addition to other tracts in patients with FoG [70, 71]. Functional connectivity studies suggest that FoG patients may have significantly stronger connectivity between the PPN and supplementary motor area (SMA) [70], possibly reflecting maladaptive compensatory mechanisms. The integrity of these tracts has not been studied in patients who have undergone PPN-DBS. The variability of this deficit in structural and functional connectivity to and from the PPN may at least partially explain the variable results within the literature. In addition, the PPN tends to be spatially diffuse in humans and microelectrode recording is not helpful intraoperatively, thus making precise lead placement difficult and potentially contributing to further variability from study to study.

The experience and results with PPN-DBS are in their infancy. More precise targeting strategies with improved technology (i.e., improved imaging and programming) are required. It remains to be seen whether PPN-DBS should be an adjunct target to STN- or GPi-DBS for better overall motor control.

#### 2.2.2. Combined Pedunculopontine Nucleus and Caudal Zona Incerta Stimulation

Khan et al. investigated the effects of bilateral PPN-DBS and caudal cZI-DBS in a blinded study of 7 patients with PD [47]. The authors demonstrated an 18.8% improvement in UPDRS III score and a 26.3% improvement in axial symptoms (items 27–30 on UPDRS III) of levodopa therapy. However, the same subscore was only significantly reduced in the “on” medication state when the PPN and cZI were stimulated in concert. This study suggested that, with these stimulation parameters, PPN stimulation alone was insufficient in improving “on” medication and resistant axial symptoms and that costimulation of cZI could provide an additive, beneficial role.

#### 2.2.3. Substantia Nigra Pars Reticulata

The SN is a dense, laterally oriented collection of dopaminergic and GABAergic neurons located within the ventral midbrain, just dorsal to the corticospinal and corticobulbar tracts, ventral to the red nuclei, and lateral to the ventral tegmental area [72]. Its 2 components, the SNr and SNc [63], have traditionally been considered the major output and input nuclei, respectively, of the basal ganglia. While there is fair overlap, the SNr lies ventrally and laterally to the SNc in the midbrain [73]. In the classically held framework of basal ganglia circuitry, facilitation of movement was felt to be achieved through activation of a direct pathway from striatum to output nuclei (SNr and GPi), while inhibition of movement occurred through excitation of an indirect pathway (through globus pallidus externus and STN) [72]; however, recent advances in modeling of striatonigral-thalamocortical pathways have made it clear that while the classical model of basal ganglia circuitry provides a solid foundation for the understanding of its complex interconnections, it hardly captures its complete intricacies [72].

The SNr is another key player in the MLR, via its significant efferent GABAergic input to the PPN [74]. Efferents from the lateral SNr to the PPN are felt to modulate postural tone, while its medial efferents projecting to the cuneiform nucleus of the MLR influence locomotion [74]. It may not then be surprising that axial motor symptomatology, including gait impairment and PI, in patients with PD has shown favorable response to SNr stimulation in the literature [55–57, 75] (Table 2). Significant improvements in UPDRS III axial motor subscores and braking capacity, but not in distal motor symptoms (segmental akinesia, limb rigidity, and tremor), have been observed previously with SNr-DBS [55]. In contrast, one of the more recent double-blind, cross-over, randomized controlled trials with combined STN and SNr stimulation did show significant improvement in FoG, but not in other axial symptoms when compared to STN-DBS alone [56]. With SNr-DBS, one should be cautious about the possibility of worsening akinesia, as increased immobility and recurrent falls were reported in 1 patient in the same study during the last week of follow-up under combined STN and SNr stimulation [56].

While some benefit from SN stimulation has been reported, significant and variable impacts on mood and behavior can occur, likely owing to its limbic projections [76, 77]. Reports of acute depression [78, 79], hypomania [77], and mania [76, 80] secondary to high frequency SN stimulation are evident in the literature. While it is difficult to rule out STN participation in the provocation of mood symptoms, it is clear that stimulation of more ventrally placed leads within the SN and likely the SNr can preferentially elicit these symptoms.

#### 2.2.4. Motor Cortex

Extradural motor cortex stimulation (EMCS) has been studied as another treatment modality in PD, particularly for those patients with advanced PD who are poor surgical candidates [81–86]. The primary motor cortex is a key component of corticobasal ganglia loops and thus forms a potential therapeutic target in PD [87]. Tremor and rigidity in PD can be suppressed by EMCS [58, 88], and benefit has been seen in advanced PD [82, 83]. Since initial reports, numerous studies have investigated the role of EMCS for the treatment of advanced PD, with variable results [58–60, 84, 86, 89–91] (Table 2).

The largest study of EMCS in 41 patients with advanced PD (not eligible for DBS) showed improvement in standing, gait, and motor performance [58], though these results were not supported by other studies [59, 60, 91]. Additional studies have shown that EMCS improved quality of life parameters and modestly reduced levodopa dose but did not improve UPDRS III scores or axial symptoms [90, 92].

#### 2.2.5. Centromedian and Parafascicular Nuclei

A single study demonstrated that CMPf stimulation alone led to significantly reduced FoG, where GPi stimulation alone did not [38]; however, this study had a sample size of only 6 patients. The authors further observed that CMPf stimulation alone may not control PD motor symptoms adequately. This observation raised the possibility of multiple-target stimulation strategy to optimize axial symptoms and overall motor control in PD.

### 2.3. Refractory Axial Symptoms-Speech and Swallowing

To date, no convincing evidence has demonstrated improvements in speech or swallowing in PD with STN- or GPi-DBS. Speech and swallowing can worsen with DBS surgery or programming. Research on the impact of cZI-DBS on associated motor symptomatology in PD has also taken place. Particular focus in the literature has been given to the effects of cZI-DBS on speech and its related domains. Stimulation of cZI was shown to have a deleterious effect on voice intensity when compared to STN-DBS [93], while articulatory precision of speech also worsens in patients receiving cZI-DBS [94]. Significant impairment in verbal fluency is also observed in the immediate postoperative period; however, this deficit does not maintain significance in the long term [95]. Speech intelligibility has been demonstrated to be significantly reduced in cZI-DBS patients speaking from a read-speech passage [96]; however, this effect was not reproduced when evaluated from spontaneous speech at 1 year postoperatively, suggesting that the impact of cZI-DBS on speech intelligibility may have initially been overstated [97]. While STN-DBS has beneficial effects on pitch variability and range, cZI-DBS displayed no such benefit in a small study of 16 patients with 1-year follow-up [98]. The effect of cZI-DBS on swallowing dysfunction has also been evaluated in 2 longitudinal, prospective studies of 8 and 9 patients [99, 100]. Both studies demonstrated that cZI-DBS did not have a clinically significant impact on either swallowing function or self-reported swallowing-specific quality of life at 1 year postoperatively. Further studies should help clarify the effect of cZI-DBS on both speech and swallowing dysfunction. In 1 study of EMCS in advanced PD, Pagni et al. demonstrated improved speech and swallowing in patients who are not DBS candidates [58].

Speech and swallowing symptoms following DBS have yet to be defined within the current literature. Methodology in assessing the symptoms varies from study to study. Severity of dysarthria/dysphagia preoperatively, duration and severity of disease, and positioning of the electrode(s) are all critical contributing factors in speech outcomes. Large-scale studies and systemic analyses are required.

## 3. Nonmotor Symptoms of PD

NMS are debilitating in PD. Robust evidence is lacking for STN- and GPi-DBS in treating NMS. A number of reports have demonstrated that PPN-DBS is capable of modulating the NMS of PD, including cognition, sleep, and attention [101–103]. The cognitive benefit of PPN-DBS has been reported in a small number of uncontrolled studies, with bilateral PPN stimulation reducing reaction time when assessing executive function and working memory and improving delayed recall and verbal fluency [101, 102]. It has been postulated that the cognitive improvement in these domains might be mediated via activation of ascending cholinergic neurons to the thalamic CMPf, subsequently leading to widespread activation via intralaminar thalamic nuclei. Indeed, functional imaging via positron emission tomography has shown an increase in fluorodeoxyglucose uptake in prefrontal areas, suggesting a modulation of thalamic metabolism after PPN-DBS [104]. Romigi was the first to identify the role of PPN-DBS in sleep, demonstrating that bilateral PPN stimulation resulted in increased rapid eye movement (REM) sleep in patients with PD [105]. Similarly, Lim et al. showed that unilateral PPN-DBS in 3 PD patients and 2 PSP patients resulted in increased nocturnal REM sleep [106]. In a subsequent study by the same group, the authors noted that bilateral, low-frequency stimulation of the PPN resulted in improved attention in 2 patients with PD [107]. No other studies to date have investigated the role of PPN-DBS in attention.

DBS targets involved in memory circuits have garnered interest in recent years. To date, only 1 human study of DBS with bilateral STN and nucleus basalis of Meynert (NBM) stimulation in PD dementia (PDD) has evaluated the potential for cognitive and/or memory improvement [108]. In this study, STN-DBS alone yielded significant improvements in motor functioning, but not in memory or cognition. The addition of NBM stimulation to STN stimulation produced significant improvements in memory and cognitive functioning, manifested as improved performance on the Rey Auditory Verbal Learning Task, Trail-Making Test A, and the Clock Drawing Test.

## 4. Discussion

A multitude of new developments have been made in the area of alternative DBS targets in PD treatment over the last two decades. Research has focused on novel DBS targets, with the aim of relieving motor symptoms and NMS that are usually refractory to dopaminergic agents and traditional STN-, GPi-, and VIM-DBS.

Stimulation of the cZI has shown promise in alleviating severe parkinsonian tremor, amongst other types, and its costimulation with PPN could provide an additive benefit on axial symptoms and PI. cZI stimulation is relatively new in its conception and additional studies are required to further evaluate its possible deleterious effects on speech, particularly voice intensity and articulatory precision.

Studies investigating axial motor symptomatology and PI with PPN stimulation have yielded mixed results. From a technical aspect, considerable variability exists amongst stimulation parameters in PPN-DBS studies (Table 1) and may account for the variable degrees of success in relieving axial motor symptoms. Additionally, the PPN tends to be spatially diffuse in humans and electrophysiological recording intraoperatively is not as helpful [109] as that of the STN, GPi, or VIM. The connectivity deficit of the PPN should also be taken into account with invasive procedures like DBS. White matter tract integrity may prove fruitful with respect to patient selection. With regard to study design, a PD population with clear dopamine-resistant gait and balance deficits should be chosen. Moreover, whether or not study subjects have concurrent STN- or GPi-DBS should be considered and studied systemically to verify the therapeutic benefit of PPN stimulation. As indicated in Table 1, few studies have been randomized and double-blinded. High quality randomized studies with standardized outcomes are needed.

The SNr represents an area of great importance in the complex hierarchy of basal ganglia circuitry and studies evaluating its potential as a DBS target have yielded mixed results. While some studies of SNr-DBS have shown improvement in axial motor symptoms, the incidence of acute mania, hypomania, and depression suggests that its utility as a target in alleviating PD symptoms may be limited by these adverse changes.

EMCS and CMPf-DBS provide some benefit in PD symptomatology. However, evidence is not conclusive for either target to be superior to STN or GPi in motor control.

NMS symptoms are disabling in PD patients. Although there is some evidence that PPN-DBS improves NMS, data are as of yet too limited to consider PPN-DBS as a therapeutic option for this domain of symptomatology. PPN-DBS may prove to be a safer target in the cognitive domain, particularly when considering the possible impact of STN- and GPi-DBS on cognition.

## 5. Conclusions

The future of DBS in PD appears promising. The field has advanced significantly with a number of new targets to address the refractory symptoms of PD. Amongst the studies investigating these novel targets, the large majority are open-label and are not powerful enough to determine true therapeutic benefit. Future, large-scale randomized studies focusing on identifying ideal candidates, optimal targets, and stimulation parameters would certainly be of utility in triggering the DBS community to perform more robust comparisons across studies.

## Figures and Tables

**Table 1:** Summary of studies on PD patients implanted with PPN-DBS for gait and balance impairment.

Study	Number of Patients	Inclusion criteria	Study design	Stimulation target	Stimulation parameters	Outcomes	Adverse events	Comments
Mazzone et al. [39]	2	FoG	Open label	Bilateral PPN	Bipolar; 10 Hz	Intraoperative improvement of UPDRS III score	NR	First human study to demonstrate the potential efficacy of PPN-DBS in PD

Plaha and Gill [40]	2	FoG, PI, and frequent falls	Open label	Bilateral PPN	Bipolar; 20–25 Hz	UPDRS improved by 53%; UPDRS III by 57%	Certain stimulation frequencies can exacerbate gait	Short follow-up, 42 days for patient 1 and 16 days for patient 2

Stefani et al. [41]	6	FoG, 3 had “on” FoG, UPDRS-III > 70, and levodopa-induced dyskinesias	Open label	Bilateral STN and PPN	Bipolar; 25 Hz at PPN	PPN stimulation showed more benefit on posture and gait items compared to STN stimulation; UPDRS III improved by 32%; axial symptoms (UPDRS 27–30) by 60%	Paresthesia	Total length of study was 6 months; noted decline in motor benefit; trend of improved UPDRS scores with both STN and PPN stimulation

Strafella et al. [42]	1	Advanced PD, FoG, PI	Open label	Unilateral PPN	70 Hz	UPDRS improved by 19%, mainly in relation to gait, tremor, and bradykinesia	NR	PET studies showed increased rCBF in different subcortical areas, most notably in the thalamus bilaterally

Ferraye et al. [43]	6	Severe FoG unresponsive to levodopa and STN stimulation	Double-blinded assessment	Bilateral STN and PPN	Bipolar; 15–25 Hz	Only FoG showed clear improvement; gait and PI scores did not improve; falls unrelated to FoG were unchanged in 5/6	Seizure in 1 patient; stimulation frequency dependent oscillopsia; paresthesias; limb myoclonus	The total length of the study was 1 year; objective improvement of FoG in 2 patients

Moro et al. [44]	6	Age < 70, severe “off” FoG and PI; no dementia	Double-blinded assessment	Unilateral PPN	Bipolar; chronic stimulation frequency of 67 Hz	Improvement in UPDRS item 13 (falling) by 75% at 3 and 12 months	Stimulation frequency dependent oscillopsia and paresthesias	First double-blinded study to investigate unilateral PPN stimulation; total study length was 1 year

Thevathasan et al. [45]	11	Severe FoG and PI, in addition to falls both in the “on” and “off” states	Open label	Bilateral PPN in 8 patients, bilateral PPN and ZI in 2 patients, and unilateral PPN and bilateral ZI in 1 patient	Bipolar; 20–35 Hz	Improvement in frequency of falls and gait	NR	Follow-up 3–38 months

Thevathasan et al. [46]	5	Severe FoG and PI, in addition to falls persisting in the “on” state	Open label	Bilateral PPN	Monopolar; 35 Hz; PPN target more caudal than previous	Improvement in all by FoG and falls questionnaire at 3 months and 2 years	Stimulation frequency dependent decline in motor function and gait; oscillopsia	Total study length was 2 years; assessments at 3 months and 2 years

Khan et al. [47]	7	PD patients with severe FoG, PI, falls during “on” and “off” states	Open label	Bilateral PPN, in combination with cZi stimulation	Bipolar; 60 Hz (PPN)	Improvement in UPDRS III score (18.8%) and axial symptoms score (26.3%)	Akinesia in 2 patients	Follow-up 12 months; similar benefit with ZI stimulation versus ZI and PPN “on”

Thevathasan et al. [48]	7	PI, severe FoG, and falls during “on” state	Open label	5 bilateral and 2 unilateral	Bipolar; 35 Hz	Improvement in freezing of gait questionnaire, turn task duration, and cadence	NR	First study to directly compare unilateral versus bilateral stimulation; less robust result in unilateral PPN

Mazzone et al. [49]	28	24 patients had PD and 4 had PSP	Open label	Both bilateral (6) and unilateral (22) PPN	Bipolar; unilateral (40 Hz) and bilateral (25 Hz)	Improvement in UPDRS III score in “off” medication and “on” stimulation	None	The largest and longest study of PPN DBS to date with a mean follow-up of 3.8 years; included patients from prior studies

FoG, freezing of gait; NR, not reported; PD, Parkinson's disease; PET, positron emission tomography; PI, postural instability; PPN, pedunculopontine nucleus; PSP, progressive supranuclear palsy; rCBF, regional cerebral; STN, subthalamic nucleus; UPDRS, United Parkinson Disease Rating Scale; ZI, zona incerta.

**Table 2:** Summary of studies on PD patients implanted with novel DBS targets (SNr, motor cortex, CMPf) for gait and balance impairment.

Study	Study subjects/inclusion criteria	Study design	Effect on FoG/PI/falls	Motor effects	Comments
*SNr*					

Chastan et al. [55]	7 patients with levodopa and STN-DBS. At least one contact of each electrode was located within the SNr	Open label	Significant improvements in UPDRS III axial motor subscores and in braking capacity	No improvement in motor symptoms (segmental akinesia, limb rigidity, and tremor)	No specific criteria for axial involvement

Weiss et al. [56]	12 patients, with combined STN and SNr stimulation; axial UPDRS ≥ 12; advance PD patients with gait/balance impairment; refractory to medical treatment	Double-blind, cross-over, randomized controlled trial	With combined STN and SNr stimulation improvement in FoG at the 3-week follow-up	No global effect on axial motor domains; no benefit for segmental motor functions	Immediate assessment and 3-week follow-up; long term effects unclear

Brosius et al. [57]	1 patient with advance PD, severe FoG; unilateral STN/SNr stimulation	Case report	Significantly improved FoG in a patient with advanced PD, using interleaving setting		Contralateral STN, the more severe side with STN/SNr

*Motor cortex*					

Pagni et al. [58]	41 patients with advanced PD; not DBS candidates, Hoehn-Yahr III–V; unilateral lead over hand area of motor cortex	Open label	Improvement in standing, gait, and motor performance; significant improvement in UPDRS axial scores	Improved “off” medication UPDRS-III; not significant for “on” medication score	Sustained improvement of quality of life measures through 3-year follow-up

Cilia et al. [59]	5 patients with advanced PD	Open label	Subjective improvement of axial symptomatology	No quantifiable clinical benefit at 6 months	Small sample size; subjective improvement only

Fasano et al. [60]	1 patient with severe PD who was unable to stand from sitting without assistance	Case report	Able to stand without assistance, with improvement in both axial akinesia and walking		Case report; short lasting benefit (5 months)

*CMPf*					

Mazzone et al. [38]	6 PD patients with disabling FoG, with GPi and CMPf-DBS	Open label	CMPf activation was more efficacious on freezing of gait	A significant amelioration of UPDRS scores was achieved	Small sample size; observation of CMPf stimulation alone may not control PD motor symptoms adequately

CMPf, centromedian-parafascicular nuclear complex; FoG, freezing of gait; GPi, globus pallidus, internal segment; PD, Parkinson disease; PI, postural instability; SNr, substantia nigra pars reticulata; STN, subthalamic nucleus; UPDRS, United Parkinson Disease Rating Scale.

## Abbreviations

cZI: Caudal zona incerta
CMPf: Centromedian-parafascicular nuclear complex
DBS: Deep brain stimulation
EMCS: Extradural motor cortex stimulation
FoG: Freezing of gait
GPi: Globus pallidus pars interna
MLR: Mesencephalic locomotor region
MPTP: 1-Methyl-4-phenyl-1,2,3,6-tetrahydropyridine
NBM: Nucleus basalis of Meynert
NMS: Nonmotor symptoms
PD: Parkinson's disease
PI: Postural instability
PPN: Pedunculopontine nucleus
PSA: Posterior subthalamic area
PSP: Progressive supranuclear palsy
REM: Rapid eye movement
SMA: Supplementary motor area
SN: Substantia nigra
SNc: Substantia nigra pars compacta
SNr: Substantia nigra pars reticulata
STN: Subthalamic nucleus
UPDRS: Unified Parkinson's Disease Rating Scale
VIM: Ventral intermediate nucleus
VOA: Ventralis oralis anterior
ZI: Zona incerta.
